# Sensitivity and Stability Enhancement of Surface Plasmon Resonance Biosensors based on a Large-Area Ag/MoS_2_ Substrate

**DOI:** 10.3390/s19081894

**Published:** 2019-04-21

**Authors:** Nak-Hyeon Kim, Munsik Choi, Tae Woo Kim, Woong Choi, Sang Yoon Park, Kyung Min Byun

**Affiliations:** 1 Advanced Institutes of Convergence Technology, Seoul National University, Suwon 16229, Korea; nh-kim@snu.ac.kr (N.-H.K.); yoonpark77@snu.ac.kr (S.Y.P.); 2 College of Electronics and Information, Dept. of Biomedical Engineering, Kyung Hee University, Yongin 17104, Korea; blue-sky031@khu.ac.kr; 3 School of East-west Medical Science, Kyung Hee University, Yongin 17104, Korea; tw1275@khu.ac.kr; 4 School of Advanced Materials Engineering, Kookmin University, Seoul 02707, Korea; woongchoi@kookmin.ac.kr

**Keywords:** surface plasmon resonance, biosensor, MoS_2_ monolayer, oxidation, sensitivity enhancement

## Abstract

Surface plasmon resonance (SPR) sensors based on a silver film suffer from signal degradation due to silver oxidation in aqueous sensing environments. To overcome this limitation, we fabricated the planar plasmonic substrate employing an atomic MoS_2_ layer on a silver surface. Successful production of a large-area MoS_2_ monolayer blocks the penetration of oxygen and water molecules. In addition, we theoretically and experimentally found that MoS_2_ layer on the silver film can improve the SPR sensitivity and stability significantly. In this study, the proposed SPR substrate has the potential to provide highly enhanced sensor platforms for surface-limited molecular detections.

## 1. Introduction

A surface plasmon (SP) is an electron charge density wave that exists at the interface between a thin metal film and a dielectric and propagates along the surface of the metal film [1]. When the transverse magnetic (TM) polarized light is incident on the metal film at a specific angle, the momentum of the incident light becomes equal to that of the plasmon and resonance occurs under this condition, which is called surface plasmon resonance (SPR). The specific angle at which resonance occurs and the reflected light gets completely attenuated is the SPR angle. When biomolecules adhere to a metallic surface, the resonance angle changes in proportion to the concentration of the target analytes [2]. Since SPR biosensors have advantages such as sensitivity, quantitative response, rapid and label-free detection, they have been widely used in a variety of analytical research fields [3].

Gold is typically used as a SPR substrate material, due to its chemical stability and reliability. On the other hand, it has been known that surface plasmons propagating along a silver surface exhibit a longer penetration depth into dielectric than those supported by a gold film [4]. SPR biosensors based on a silver film produce a sharp SPR curve, which can provide high selectivity and sensitivity in SPR imaging detection. However, an inevitable problem associated with application of silver films in SPR biosensors is that silver is highly susceptible to oxidation [5]. In particular, the oxidation of silver film can be fatal when it is exposed to an aqueous medium. The silver oxide formed by the oxidation process can degrade the SPR signal and interrupt surface-limited biomolecular reactions. In order to avoid silver oxidation, several approaches using stable metallic or dielectric coatings have been proposed. For example, addition of thin gold overlayer can prevent a silver film from being oxidized. [6,7]. However, individual surface plasmons produced by gold and silver films may interfere with each other, leading to a notable sensitivity degeneration compared to the case of a conventional single metal film [8].

Since the discovery of the single atomic layer two-dimensional (2D) structure of graphene, it has been considered a potential candidate for the protective layer of SPR substrates [9,10,11]. The thickness of a single graphene layer is about 0.34 nm and molecules cannot pass through its ring structure due to the high electron density of the hexagonal rings [12]. Hence, graphene is impermeable to oxygen and effective for protecting metal surfaces against corrosion [13]. Moreover, the plasmonic effects of graphene have been demonstrated for biological and chemical sensing applications both in the theoretical and experimental studies [14,15].

Recently, monolayers of molybdenum disulfide (MoS_2_) that belongs to the transition-metal dichalcogenides (TMDC) have been gaining great attention. The 2D structure of MoS_2_ is stacked in the vertical direction via Van der Waals forces [16,17]. As a monolayer of MoS_2_ possesses a higher optical absorption efficiency (~5%) than that of graphene (2.3%) [18,19], it can promote plasmon excitation through an efficient charge transfer between MoS_2_ and the thin metallic film. However, despite its advantages, so far it has not been possible to successfully deposit a single layer of MoS_2_ uniformly on a large surface area. In this study, we fabricated MoS_2_ monolayers by chemical vapor deposition (CVD) and transferred them onto a large-area silver substrate. By comparing the sensor performance via non-specific binding experiments, we intend to demonstrate an enhancement of sensitivity and stability of SPR substrate with no concerns about oxidation.

## 2. Materials and Methods

### 2.1. Fabrication of MoS_2_/Ag-Based SPR Sensor Substrate

**Figure 1:**
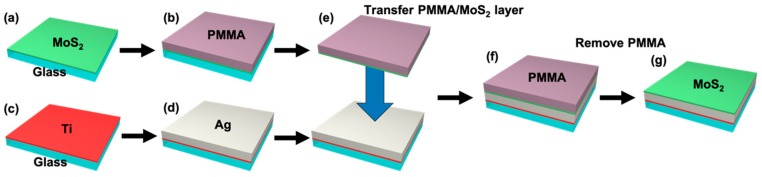
Schematic of fabrication processes of the Ag/MoS_2_ SPR sensor substrate. (**a**) MoS_2_ deposition using CVD, (**b**) PMMA spin-coating, (**c**) separation of PMMA/MoS_2_ from Sapphire glass using KOH, (**d**) Ti/Ag deposition using e-beam evaporation, (**e**) PMMA/MoS_2_ transferring to Ti/Ag substrate, (**f**) PMMA removal using acetone.

Figure 1 shows the fabrication processes of the Ag/MoS_2_-based SPR substrate. First of all, a sapphire glass (Schott, Mainz, Germany) and an NSF10 glass (Schott, Mainz, Germany) were prepared for the MoS_2_ transfer process. The glass substrates were cleaned by sonication with isopropyl alcohol for 10 min, rinsed with deionized water for 10 min, and dried with nitrogen gas for 10 min. Then, a MoS_2_ layer was deposited onto the sapphire glass substrate using a CVD process. For MoS_2_ formation, 15 mg of MoO_3_ (99.98 %, Sigma-Aldrich, St. Louis, MO, USA) and 1 g of S (99.98 %, Sigma-Aldrich, St. Louis, MO, USA) powders were used as precursors. 

The precursors were put on zone 1 of the CVD chamber and the sapphire glass substrate was put on zone 2. Then zone 1 and zone 2 were heated to the temperatures of 700 °C and 600 °C, respectively, and the pressure of the CVD chamber was maintained below 0.5 torr during 30 min for MoS_2_ deposition. After that, the temperature of the CVD chamber was decreased slowly to room temperature [20]. 5-nm thick titanium and 45-nm thick silver layers were sequentially deposited with a deposition rate of 3 Å/s onto the NSF10 glass substrate using an electron beam evaporation. The titanium layer acts as an adhesion layer for silver deposition on the NSF10 glass substrate. To transfer the MoS_2_ layer onto the silver film, PMMA was spin-coated onto the MoS_2_ layer. After PMMA deposition, the sapphire glass was removed using wet etching with KOH for 1 h, and then PMMA/MoS_2_ layers were transferred onto the silver film. Finally, the fabrication of Ag/MoS_2_-based SPR substrate with a large area was completed through PMMA removal by using a wet etching process with acetone.

### 2.2. Optical Setup and Experimental Methods

Our SPR sensor system based on the Kretschmann configuration which consists of a polarized He–Ne laser (05-LHP-991, Melles Griot, Irvine, CA, USA), dual rotation stages (SR50CC, Newport, Irvine, CA, USA) with a wide scanning range of 30 to 80 degrees at a resolution of 0.002 degree, a semicircular prism (customized model, Korea Electro-Optics, Bucheon, Korea) and a photodiode (918D-SL-OD3, Newport).

We investigated the oxidation stability and sensor sensitivity of the fabricated SPR substrates. To demonstrate oxidation stability, Ag/MoS_2_ and bare Ag substrates were exposed under slow and fast oxidation conditions. Fast oxidation experiments were performed when a TM-polarized laser with a 10 mW power at the wavelength of 633 nm was incident under resonance conditions. In addition, to compare the SPR sensing performances of the fabricated substrates, immunoglobulin G (IgG) from human serum (PN I4506, Sigma-Aldrich) was used as a target analyte. Since the MoS_2_ and Ag surfaces are chemically different, a strategy to attach a non-specific analyte based on physisorption binding to the surfaces of two sensor substrates was used. IgG (200 uL, 600 nM) dissolved in a pH 7.4 phosphate buffered saline (PBS) solution, was injected through the microfluidic channel for 10 min to attach analytes onto the substrates. The substrates were cleaned and rinsed with distilled water for 5 min and then, PBS (1 mL) was injected through the fluidic channel for 5 min. The SPR signals were measured five times after each process in order to check reproducibility.

## 3. Results and Discussion

First, we confirmed that the MoS_2_ monolayer was successfully transferred to the silver substrate by analyzing the material composition of the fabricated substrate. While atomic force microscope (AFM) and energy dispersive X-ray spectroscopy (EDS) are generally used for thickness measurements and surface composition analysis, those methods cannot be used at the same time. On the other hand, Raman spectroscopy, which measures inelastic scattering photons generated by the unique vibrational spectrum of the molecule, can give us compositional information about the target sample. In addition, since the vibration mode of MoS_2_ changes as the number of MoS_2_ layers increases, the thickness can be estimated by analyzing its Raman signal. MoS_2_ has four types of vibrational modes, E^2^_2g_, E^1^_g_, E^1^_2g_ and A_1g_. The wavenumber of each mode is 32 cm^−1^, 286 cm^−1^, 383 cm^−1^ and 408 cm^−1^. Note that, wavenumber gap between E^1^_2g_ and A_1g_ can be used to find the number of MoS_2_ layers.

**Figure 2:**
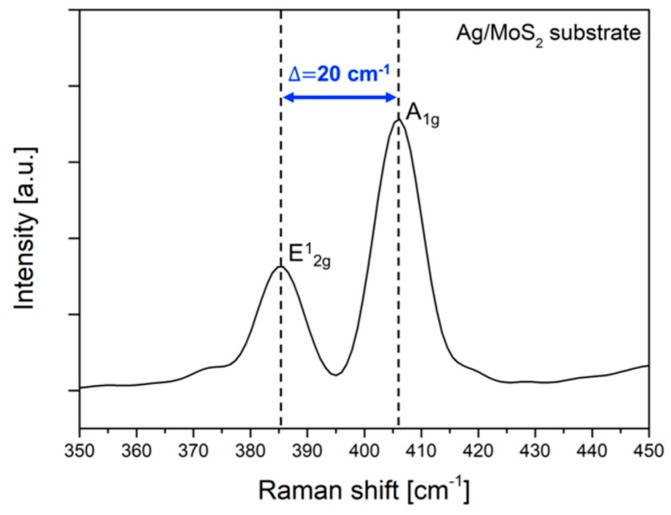
Raman spectra of the surface of Ag/MoS_2_ substrate. To confirm the number of MoS_2_ layers, wavenumber gap between E^1^_2g_ and A_1g_ mode is obtained. The measured Raman peaks are 386 cm^−1^ and 406 cm^−1^ (i.e., Δ = 20 cm^−1^), supporting that the fabricated MoS_2_ layer on the Ag substrate is a single layer.

With an increment of the layer number of MoS_2_, the wavenumber of A_1g_ mode, which is related with vertical vibration, is increased and the wavenumber of E^1^_2g_ mode, which represents vibration in the same plane, is decreased by means of Van der Waals forces and Coulombic interactions. As a result, the MoS_2_ monolayer has a wavenumber gap of 18~20 cm^−1^ and the multi-layered MoS_2_ has a wavenumber gap of 25 cm^−1^ or more [20]. Figure 2 shows the Raman spectra of the fabricated Ag/MoS_2_ substrate. The observed Raman spectra include Raman peaks of MoS_2_ at 386 cm^−1^ and 406 cm^−1^, thus Δ = 20 cm^−1^, implying that MoS_2_ monolayer was successfully transferred onto the silver substrate. In addition, no Raman peak of PMMA is found because PMMA was removed completely through wet etching process.

**Figure 3:**
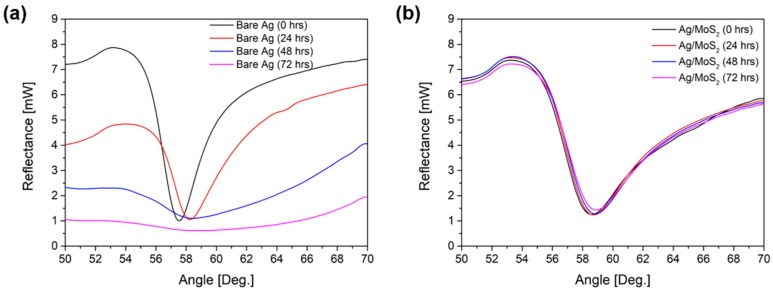
SPR signals of (**a**) bare Ag and (**b**) Ag/MoS_2_ substrates in water. Silver oxidation may cause a broader SPR dip while Ag/MoS_2_ substrate is stable due to impermeability to oxygen.

Next, we verified the role of MoS_2_ as a protective layer for silver film using slow and fast oxidation experiments. Figure 3a,b present time-varying SPR curve changes for bare Ag and Ag/MoS_2_ substrates in an aqueous solution. Since Ag_2_O is formed on the Ag surface as the oxidation progresses, the SPR signal becomes gradually broader according to the exposure time in the solution. However, the SPR signals of the Ag/MoS_2_ substrate did not vary significantly for more than four days. The MoS_2_ layer consists of a sandwich structure of two S layers and one Mo layer. These Mo and S layers have a hexagonal structure and Mo atoms are bonded with S atoms in a trigonal prism geometry. The S layers surrounding the Mo layer interacts with oxygen to give rise to high-energy barriers, which makes oxygen penetration into the MoS_2_ monolayer very difficult [21].

**Figure 4:**
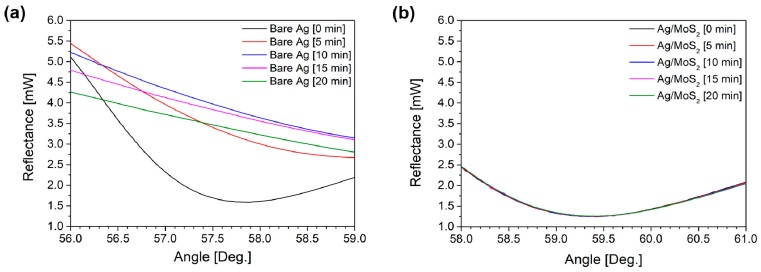
SPR signals of (**a**) bare Ag and (**b**) Ag/MoS_2_ substrates in water with laser irradiation. Bare Ag substrate is easily oxidized as soon as it is exposed to a laser light and the SPR curves drastically change. Photosensitive Ag_2_O, which is formed on the Ag surface during the progress of oxidation, decomposes when it is heated and the surface structure is damaged during the formation and decomposition of Ag_2_O.

Figure 4a,b show the SPR signal changes of bare Ag and Ag/MoS_2_ substrates when they are irradiated by a laser in an aqueous solution. Due to temperature elevation via absorption of laser energy, faster oxidation process may occur. The bare Ag substrate in Figure 4a is oxidized as soon as it is exposed to a laser light. Moreover, as the obtained Ag_2_O on a silver film is photosensitive, the oxide layer decomposes when it is heated above the threshold temperature. It is found that formation of silver oxide and its decomposition can modify the SPR curves of the substrate drastically. On the other hand, the Ag/MoS_2_ substrate does not change regardless of the laser irradiation, which means that MoS_2_ layer prevents the oxidation of silver and allows more stable features for application in practical SPR biosensors in aqueous medium.

**Figure 5:**
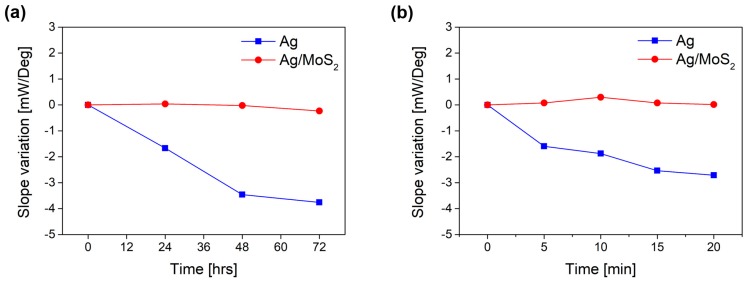
The slope variation of SPR signals of bare Ag SPR substrate (blue square) and Ag/MoS_2_ SPR substrate (red circle) under (**a**) slow and (**b**) fast oxidation conditions. The slope variation of the SPR signal, which is defined as the intensity change of reflected light according to the angle, are represented according to measurement time.

Figure 5 presents quantitatively the deformation of SPR graphs in Figure 3 and Figure 4 according to the measurement time. The slope variation in Figure 5 means the variation of maximum slope value of individual SPR curves, i.e., the difference between the initial maximum slope value and the time-varying maximum slope value. The slope variation of the Ag/MoS_2_ substrate is almost constant, however, that of the bare Ag substrate gradually decreases. This implies that a MoS_2_ layer confers enhanced stability to the substrate surface under slow and fast oxidization conditions.

**Figure 6:**
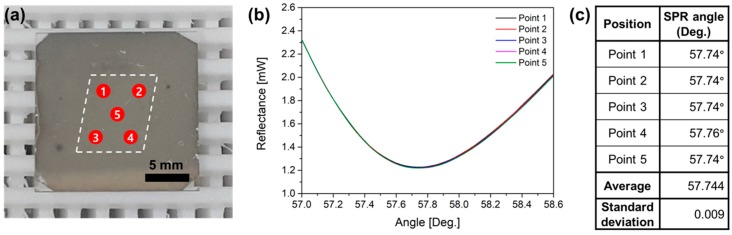
(**a**) The fabricated Ag/MoS_2_ sample and five SPR measurement points in red circles. (**b**) SPR curves of the Ag/MoS_2_ sample obtained at the five different positions. (**c**) Summary table of SPR angles and average value.

In addition, to demonstrate the spatial uniformity of the MoS_2_ layer, SPR characteristics in multiple points are shown in Figure 6. The dashed line in the middle of the sample represents the area where MoS_2_ film is formed. It is presented in Figure 6b that the SPR signals obtained at the center and four corners are very similar in shape. SPR angles and average value are summarized in the table in Figure 6c. Considering the angle scanning resolution of 0.01 degree, we confirm that the MoS_2_ layer was uniformly deposited over the surface area.

Finally, we compared the two types of SPR substrates in terms of sensor sensitivity. SPR signals of two chemically different Ag and Ag/MoS_2_ surfaces are measured through physisorption binding of IgG at a single concentration of 600 nM. Note that sensor sensitivity can be obtained by computing the change in resonance angle of two cases of with and without binding of IgG, while sensing experiments at different concentrations are possible only under chemical binding event conditions.

**Figure 7:**
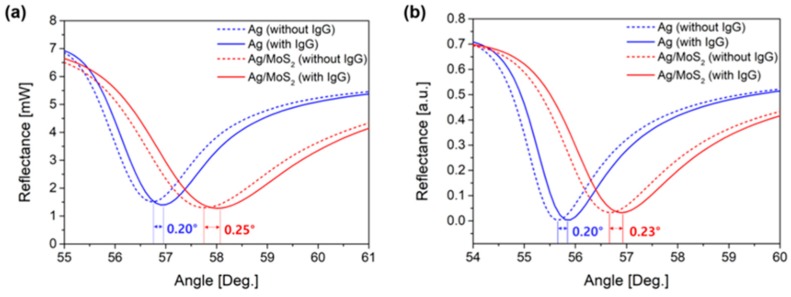
Experimental and simulation results of SPR signals for Ag and Ag/MoS_2_ substrates for IgG detection. (**a**) SPR angle shift before and after the binding reaction of IgG of Ag substrate is 0.20° and that of the Ag/MoS_2_ substrate is 0.25°. (**b**) RCWA calculation result of the SPR angle shift is 0.20° for a bare Ag substrate and 0.23° for Ag/MoS_2_ substrate.

Figure 7a shows that the SPR angle shift of the Ag/MoS_2_ substrate is larger before and after non-specific binding reaction of IgG than that of a bare Ag substrate. SPR angle shift of the bare Ag substrate is 0.20° with a standard deviation (SD) of 0.008° and that of the Ag/MoS_2_ substrate is 0.25° with SD of 0.010°, respectively. Together with an improvement of sensor sensitivity up to 125% by introducing a single MoS_2_ layer, it is expected that enhanced detection limit and responsivity are possible. It seems that the improved sensor performance is associated with the plasmon enhancement through high optical absorption efficiency of MoS_2_ layer and the promotion effect of the excitation process of an efficient charge transfer between MoS_2_ monolayer and thin metal film [22].

In Figure 7b, we show the SPR angle shift of 0.20° for a bare silver substrate and 0.23° for Ag/MoS_2_ substrate using RCWA calculation and the experimental results match the simulations consistently. The optical constants ε=(n,k) of NSF10 glass, thin layers of titanium, silver, and MoS_2_ are set to be (1.723, 0), (2.047, 3.164), (0.144, 3.81), and (5.9, 0.8) respectively, at the wavelength of 633 nm. Also, the refractive index of a phosphate buffered saline (PBS) solution is assumed to be 1.33. IgG binding reaction occurring at the surface of each sample is modeled as a 15 nm thick layer and the refractive index of the binding layer is set to be 1.35 which is determined by the effective-medium approximation according to the Maxwell-Garnett theory [23].

## 4. Conclusions

In this study, we fabricated a SPR substrate by incorporating an atomic MoS_2_ layer on top of a Ag film and demonstrated its enhancement of sensitivity and stability. In the SPR substrate based on Ag film, the exposed Ag layer was easily oxidized and Ag_2_O was decomposed by incident light in an aqueous environment. However, we found that when the MoS_2_ layer is introduced as a protective layer, the atomic MoS_2_ monolayer completely suppressed the oxidation of Ag film. The experimental results indicated that MoS_2_ monolayer can provide a reliable surface stability as well as an improved detection sensitivity. This seems to be attributable to the high energy barrier and high light absorption efficiency of the MoS_2_ monolayer. While the sensing experiment in this study is based on non-specifically bound analytes, in future research we will use a well-designed chemical binding event and the proposed Ag/MoS_2_ substrate of high sensitivity and stability is expected to be applicable to the analysis of a variety of small target molecules.
